# Artificial intelligence and internet of things oriented sustainable precision farming: Towards modern agriculture

**DOI:** 10.1515/biol-2022-0713

**Published:** 2023-10-14

**Authors:** Amit Sharma, Ashutosh Sharma, Alexey Tselykh, Alexander Bozhenyuk, Tanupriya Choudhury, Madani Abdu Alomar, Manuel Sánchez-Chero

**Affiliations:** Institute of Computer Technologies and Information Security, Southern Federal University, Taganrog, 347922, Russia; Chitkara University Institute of Engineering and Technology, Chitkara University, Punjab, India; School of Computer Science, University of Petroleum and Energy Studies, Dehradun, India; Symbiosis Institute of Technology, Symbiosis International University, Pune, Maharashtra, 412115, India; Department of Industrial Engineering, Faculty of Engineering – Rabigh, King Abdulaziz University, Jeddah 21589, Saudi Arabia; Universidad Nacional de Frontera, Sullana, Perú, Facultad de Ingeniería de Industrias Alimentarias y Biotecnología, Sullana, Peru

**Keywords:** IoT, artificial intelligence, methanol, renewal energy, sustainable development

## Abstract

Agriculture encompasses the study, practice, and discipline of plant cultivation. Agriculture has an extensive history dating back thousands of years. Depending on climate and terrain, it began independently in various locations on the planet. In comparison to what could be sustained by foraging and gathering, agriculture has the potential to significantly increase the human population. Throughout the twenty-first century, precision farming (PF) has increased the agricultural output. precision agriculture (PA) is a technology-enabled method of agriculture that assesses, monitors, and evaluates the needs of specific fields and commodities. The primary objective of this farming method, as opposed to conventional farming, is to increase crop yields and profitability through the precise application of inputs. This work describes in depth the development and function of artificial intelligence (AI) and the internet of things (IoT) in contemporary agriculture. Modern day-to-day applications rely extensively on AI and the IoT. Modern agriculture leverages AI and IoT for technological advancement. This improves the accuracy and profitability of modern agriculture. The use of AI and IoT in modern smart precision agricultural applications is highlighted in this work and the method proposed incorporates specific steps in PF and demonstrates superior performance compared to existing classification methods. It achieves a remarkable accuracy of 98.65%, precision of 98.32%, and recall rate of 97.65% while retaining competitive execution time of 0.23 s, when analysing PF using the FAOSTAT benchmark dataset. Additionally, crucial equipment and methods used in PF are described and the vital advantages and real-time tools utilised in PA are covered in detail.

## 1 Introduction

Traditional agriculture has evolved through time into a completely different form that delivers higher benefits for human life thanks to human collaboration and touch. The billion-plus population of the world is fed through agriculture, which is regarded as the oldest and most fundamental industry in the world [1]. Currently, the Gross Domestic Products of numerous nations depend on agricultural product production, in addition to technological services and crude oil, underscoring its importance as a significant industry. The majority of manual labour tasks in agriculture have been largely replaced by machines and technology over time, enhancing overall value, effectiveness, and enticing more individuals to rely on farming for their living [2]. In the upcoming years, there will be a sharp decline in arable land due to urbanisation, casting doubt on the feasibility of meeting the need to support agricultural food production. Contrary to this, it is clear from the most recent research that in order to feed the expanding global population by the year 2050, the current agricultural food output must be boosted by more than 70% [3]. Fulfilling the demand for agricultural food production is a growing concern due to a number of factors, including the decline in arable terrain, the need for physical labour, and the rising capital expenses. In order to overcome these difficulties in the long run, there is a fantastic opportunity for academia and research and development groups to come up with creative solutions that will allow them to enhance the quantity of high-quality harvest while reducing resource consumption. Some examples of such technologies are the Internet of things (IoT), artificial intelligence (AI), cloud, edge and fog computing, and 5G communication systems which are being deployed today with the objective of increasing yield and quality. Because of the advantages these technologies offer to farmers, their adoption has become a major trend in recent years. Also, this successful fusion of technology has cleared the way for the growth of “smart agriculture,” which refers to the use of smarter technologies in agriculture with the intention of increasing the efficiency of farming chores [4,5].

In the application of modern agriculture, poor preparation, ineffective harvesting, uneven irrigation, and changeable climate circumstances like droughts and floods are the main issues keeping planters from achieving their targets. These issues can be resolved by implementing AI to help farmers make informed decisions [6]. Poor agricultural results and unmet potentials can occasionally cause tension and suffering for ranchers, and they may even experience suicidal thoughts and ultimately lose their life, as is a fact in the majority of developing nations, such as Sri Lanka, India, and Bangladesh [7]. Thus, it can also cause social unrest and have an impact on a nation’s economy, as was amply demonstrated due to the economic and supply calamity that Sri Lanka experienced in 2022, which led to the government’s decision to forbid the import of any chemical fertilisers [8]. The COVID-19 global pandemic, a lethal virus epidemic that is still widespread, created supply chain and logistics problems that have significantly hampered agricultural food production in recent years [9]. Moreover, the danger of food insecurity has grown as a result of the ongoing fighting in the Black Sea region as well as supply chain disruptions in the agricultural commodities market.

### 1.1 Objectives of the study

**Figure 1:**
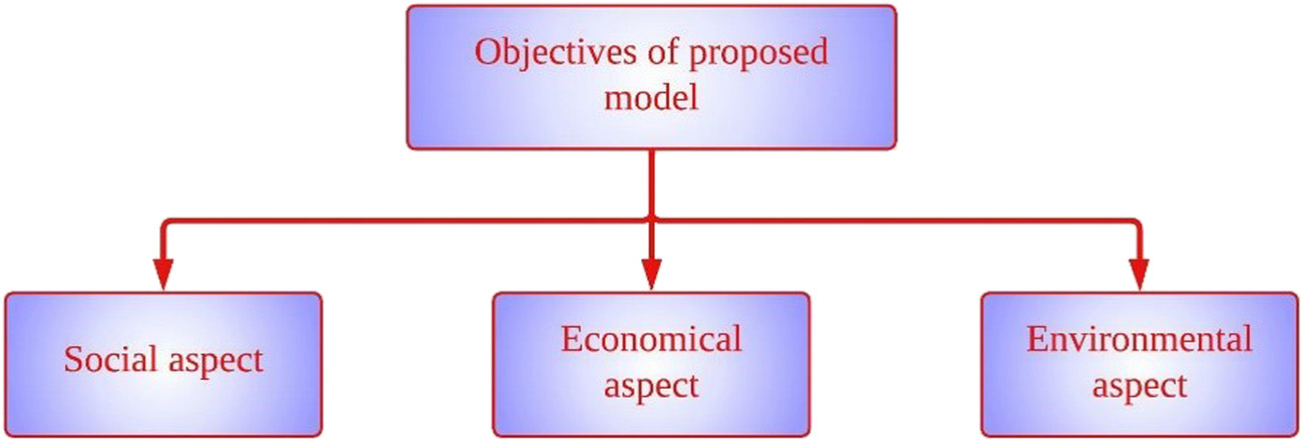
Objectives of the proposed PA model.

The basic idea is that decision-making is the main emphasis of precision agriculture (PA) when it comes to management strategy. The primary objective of the proposed model is to enhance production management and reduce environmental effect, particularly considering variable climatic conditions of today’s life. With the aim of delivering benefits such as social, economic, and environmental, this type of agriculture necessitates the use of contemporary information technology and technical solutions. The objectives of the proposed model for PA are illustrated in Figure 1.

The objectives are framed considering social, economic, and environmental aspects. The social aspect consists of safe and healthy food, saving natural resources, educational importance for rural areas, pollution reduction, and improving living standards. The economic aspect consists of minimising financial risk, optimising production efficiency and quality, and maximising gross margin. The environmental aspect consists of pollution reduction, optimal input quantities, and minimising environmental impact. Making measurements of critical factors like temperature, humidity, soil moisture, and acidity, as well as reducing the usage of pesticides and fertilisers, are the main objectives for the development of PA in order to increase yields and improve product quality. The needs of the economic and environmental elements must be met.

### 1.2 Contribution

Precision farming (PF) or PA, which uses transformative data-driven methodologies, analysis tools, and high-tech equipment to maximise crop yield, has emerged as a transformational strategy in modern agriculture. In this article, we examine the uniqueness and value of a ground-breaking method for sustainable PF that combines AI with the IoT. By combining these technologies, we aim to improve decision-making procedures, boost resource management, and advance sustainable agriculture practices by merging these technologies. Some of the contributions of the proposed approach are listed below.

#### 1.2.1 Nutrient management and water quality

This approach’s emphasis on nutrient management and water quality is one of its major contributions. The suggested methodology seeks to protect water quality by working with farmers to manage fertilisers properly. Farmers may get precise information on soil nutrient levels through installing sensors at key depths and monitoring the deployment site in real-time. With the use of this knowledge, they may apply precise nutrient management approaches, reduce nutrient runoff, and ensure fertilisers are used effectively.

#### 1.2.2 PF for efficient resource management

PF uses AI and IoT to manage resources efficiently, reducing waste and increasing production. The proposed model embraces the convergence of AI, machine learning (ML), and IoT to optimise PF practices. Real-time information on soil moisture, temperature, and nutrient concentrations may be gathered by placing IoT sensors throughout the agricultural landscapes. Farmers may then use this information to influence their decisions about irrigation, fertilisation, and pest management thanks to the AI algorithms that process it. The innovation is in the seamless fusion of AI with IoT, which results in a holistic system that optimises resource allocation and minimises environmental effect.

#### 1.2.3 Integration of emerging technologies

The scalability, efficacy, and resilience of the proposed model are further improved by integrating new technologies such as edge AI and edge intelligence. Edge AI makes it possible to analyse data at the network’s edge, which lowers latency and improves the ability to make decisions in real time. Instead of depending entirely on centralised cloud-based processing, edge AI includes doing AI computations at the network’s edge, nearer the data source. Real-time data processing and analysis are made possible by putting AI capabilities closer to the IoT sensors installed in the agricultural landscapes. This results in quicker response times and better decision-making. Edge intelligence also offers cutting-edge automation and analytics capabilities at the edge, enabling intelligent and autonomous agricultural operations. By ensuring smooth AI integration with IoT systems, farmers will have access to strong tools to monitor and improve their agricultural operations.

#### 1.2.4 IoT sensor networks for smart agriculture computation

To achieve the needed degree of computation and data collection, the use of IoT sensor networks in PF is essential. The suggested strategy makes use of sensor-based IoT networks to collect a variety of information about different agricultural characteristics including soil moisture, temperature, and nutrient levels. With the help of this extensive data, farmers can get a complete picture of their fields, spot trends, and choose wisely between irrigation, fertilisation, and crop health management. PF is revolutionised by the use of IoT sensor networks, which deliver precise, timely, and useful data for bettering agricultural practices.

**Figure 2:**
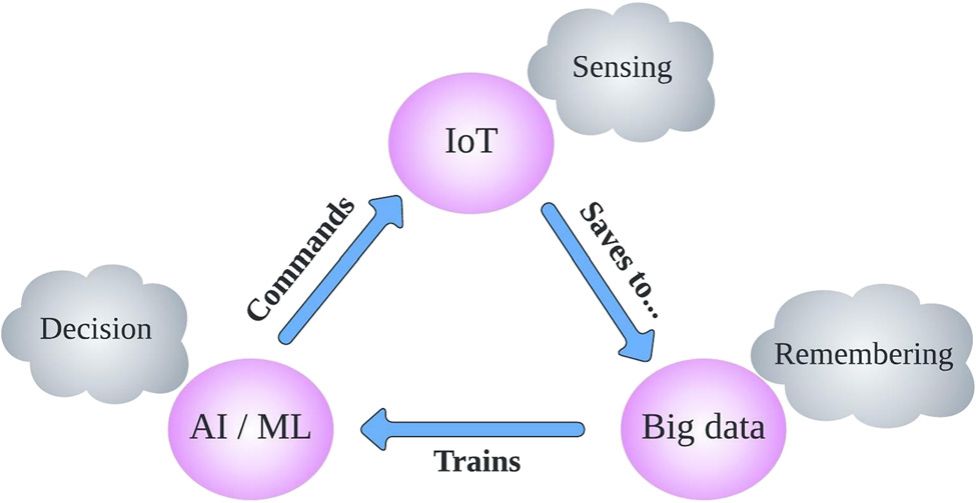
Theme of the proposed model for PA.

The theme of the proposed model for PA is depicted in Figure 2, which encompasses the latest trends in AI, ML, and IoT. The model integrates IoT-enabled solutions that leverage AI and ML interfaces through the internet. IoT and sensors provide vast amounts of data, which can be further processed by AI and ML to identify patterns, address data inconsistencies, and predict future outcomes.

The application of IoT in smart agriculture and PF allows for advanced computation, and the integration of AI and ML further strengthens this model. Our proposed approach embraces the latest developments in IoT sensor-based networks and leverages emerging technologies. In order to contribute to the cutting-edge field of smart agriculture, our suggested strategy incorporates the most recent advancements in IoT sensor-based networks and makes use of upcoming technology.

The novelty of the AI and IoT-oriented sustainable PF approach lies in its integration of AI, ML, and IoT in sustainable PF. By leveraging these cutting-edge technologies, the model offers real-time monitoring, data-driven decision-making, efficient resource management, and environmental sustainability. It empowers farmers to adopt PF practices that optimise crop production, minimise environmental impact, and ensure long-term agricultural viability. Through the strategic deployment of IoT sensor networks and the application of AI and ML algorithms, farmers can collect and analyse data on soil moisture, temperature, and nutrient levels, enabling informed decision-making. The integration of edge AI and edge intelligence further enhances scalability and effectiveness, enabling real-time data processing and intelligent automation. This innovative approach marks a new era of smart and sustainable farming, where advancements in AI, ML, and IoT hold boundless potential for revolutionising agriculture and addressing the challenges faced by the industry.

### 1.3 Organisation of the study

The structure of the article is as follows. In Section 2, which comes after the introduction, we highlight the most recent findings in the area while setting our work apart from theirs. Section 3, describes the methodology of the proposed model of PA. Based on an experimental assessment of our investigation, Section 4 then highlights our research approach and followed which is a discussion in Section 5. The conclusion of the study includes the findings from our investigation which is presented in Section 6.

## 2 Related work

PF refers to the precise application of agricultural inputs relative to crop, soil, and weather in order to enhance agricultural quality, profitability, and output. PA is a modern agricultural practise that employs technologies such as remote sensing, Geographical Information System (GIS), and Global Positioning System (GPS) to increase profitability and productivity [10]. It enables producers to use crop inputs such as irrigation water, pesticides, and fertilisers more efficiently. Utilising inputs more efficiently will increase crop yield and quality without contaminating the environment, leading to sustainable agriculture and sustainable development. Utilising new technologies and collected field data, PA entails doing the right thing, at the right time, in the right location. The collected data can be used to evaluate optimal sowing density, estimate fertiliser and other input requirements, and predict crop yields more precisely [11]. It reduces the use of inputs such as fertilisers, pesticides, labour, and water, and guarantees higher quality crops, regardless of local soil and climate conditions. PA, Satellite farming, or site-specific crop management are commonly defined as “a technology-enabled approach to farming management that observes, measures, and analyses the needs of individual fields and crops.” According to McKinsey, two trends [12] are shaping the development of PA:

1. Big data and sophisticated analytics capabilities.
2. Robotics that offers aerial imagery, intelligent sensors, and complex local weather forecasts.

Adopting PF reduces production costs and waste because each plot’s specific requirements are met. PF is practised through the use of analytical software and technological instruments. By deploying sensor-equipped devices throughout the fields, rigorous data are collected on soil testing, allotment measurement, analysis of weather patterns, and crop analysis. The data are calibrated in order to draw conclusions, and a very detailed and precise set of practises can be adopted based on these results.

A number of research projects on many elements of agriculture have already been carried out in recent years with the application of ML in PF. Consequently, we provide a tabular summary of the most recent study (Table 1) to provide a better overview and to set our work apart from theirs. The next major agricultural revolution is PF, also referred to as PA. It intends to provide farmers with real-time information about their farms and livestock as needed, enabling them to make precise decisions in a timely manner that will increase yield and reduce waste of limited resources [23,24]. The level of soil fertiliser, water requirements, soil nutrient level, and animal health are just a few of the environmental data connected to farms and livestock that are collected by a variety of IoT sensors in PF [25,26]. In order to increase the farm’s overall efficiency, the data may also provide useful insights to farmers when it has been properly examined and polished [27,28]. These insights could include information about the health of crops, animal and plant illnesses, and weather patterns. The precise deployment of PF applications can benefit the entire crop cycle. At the moment, PF solutions are heavily used to boost productivity and optimise crop production. The total market value for PA solutions has almost doubled since 2016, according to Libelium, a leading provider of IoT technological solutions for smart agriculture and other relevant IoT sectors. The most recent research shows that, with the current rate of technological advancement, AI will affect the world more than anything else in human history [29,30]. Since AI is the foundation of PF, it is presently used in numerous applications that help farmers take quick action. IoT sensors and UAVs generate millions of data points every day on an average farm, amassing a significant amount of data also known as big data [31]. Majority of the time, these large data will be uploaded to the cloud, where AI will be employed to deduce its meaning [32,33]. In particular, PF seeks to take use of the substantial spatial and temporal variability of crop and environmental variables in order to customise management in a clear and comprehensive way. In this proposal, how to deal with variations in yield potentials, topography, soil qualities, nutritional requirements, and abiotic (like weather) and biotic stresses (like pest and weed infestation) are discussed. Through PF, the correct decision can be made in the right place, at the right time, and in the right way. Farmers can employ several (combinations of) technology to achieve this. In this project, we will initially distinguish diagnostic technologies for collecting or generating information and applicative technologies for implying altered management actions. Data collection and data structuring are the basic building blocks of PF, but in the end, the high potential of PF results from the application of a variety of technologies to generate management practises from the obtained data [34,35,36].

**Table 1:** Overview of most recent studies with their applications and contributions

References	Technology used	Application	Contribution
[13]	Naïve Bayes, random forest	Crop supervision	The study suggested developing web-based and intelligent mobile applications that would use data mining techniques to recommend what crops to grow, crop rotation, and fertiliser identification
[14]	Support vector machine, decision tree	Crop supervision	The authors presented a recommendation method for crop compatibility and pest control that is powered by ML. In addition, they discovered that SVM, as opposed to other ML algorithms, produced the greatest results
[15]	Support vector machine, neural network	Soil Supervision	In order to categorise datasets from diverse places based on the soil qualities, the authors conducted an empirical analysis of several data mining classification techniques
[16]	Neural network	Soil supervision	For each kind of soil, the authors offered a detailed recommendation methodology while taking into account the various features of the soil
[17]	Artificial neural network	Soil and crop supervision	The authors created a mechanism to propose crops for farms depending on the soil conditions there, and then trained their underlying ML models using data from a soil sampling lab
[18]	Neural network	Crop supervision	The scientists described a method for a near-sensor neural network that can detect the Codling Moth in apple orchids automatically. The device automatically identifies the kind of bug it has spotted and sends out alerts to the farmers as soon as possible
[19]	Random forest	Crop supervision	For variable rate pesticide spraying, the scientists devised real-time, AI-powered crop/weed identification
[20]	Random forest	Crop supervision	To suggest crops based on site-specific factors, the authors proposed a majority-voting ensemble model
[21]	Decision tree	Water supervision	A real-time smart irrigation system was proposed by the authors, that would notify ranchers in real-time when to irrigate their fields using the DT ML algorithm
[22]	ZeroR, OneR	Water supervision	The authors automated the extraction of new knowledge in the form of generalised decision rules for the best use of natural resources, such as water in agricultural land, using ML approaches

## 3 Methodology

**Figure 3:**
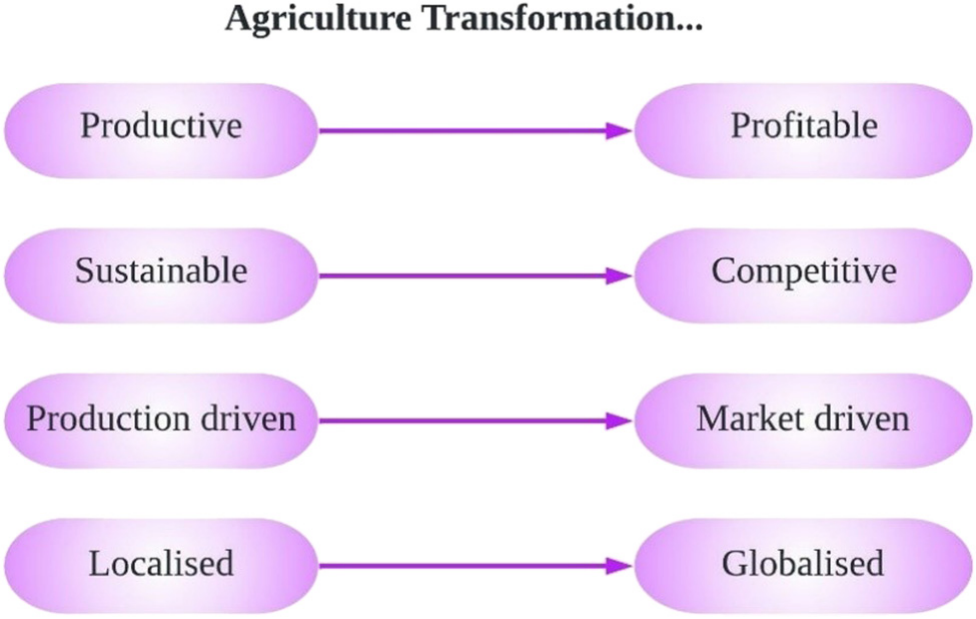
Agricultural transformation concept.

The primary objective of the proposed PA model is agricultural transformation which is depicted in Figure 3. Three main goals of proposed model of PF are Profitability, Efficiency and Sustainability. Profitability refers to recognising crops and marketing strategically as well as prefiguring Return on Investment (RoI) based on cost and gross profit.

The goal of this proposed model is to develop a methodology for identifying the causes of within field variation in crop performance. Efficiency can be improved by implementing precise algorithms, leading to rapid and cost-effective farming opportunities. This allows for more efficient overall resource utilization and the development of practical guidelines required to implement PF technology for better management. Sustainability refers to better environment and socio-economic operation assures additive improvements in each season for all the performance indicators. To explore the possibilities of using remote-sensing methods and GIS for enabling real-time management decisions during crop growth [37,38,39].

### 3.1 Strategy and methodology

**Figure 4:**
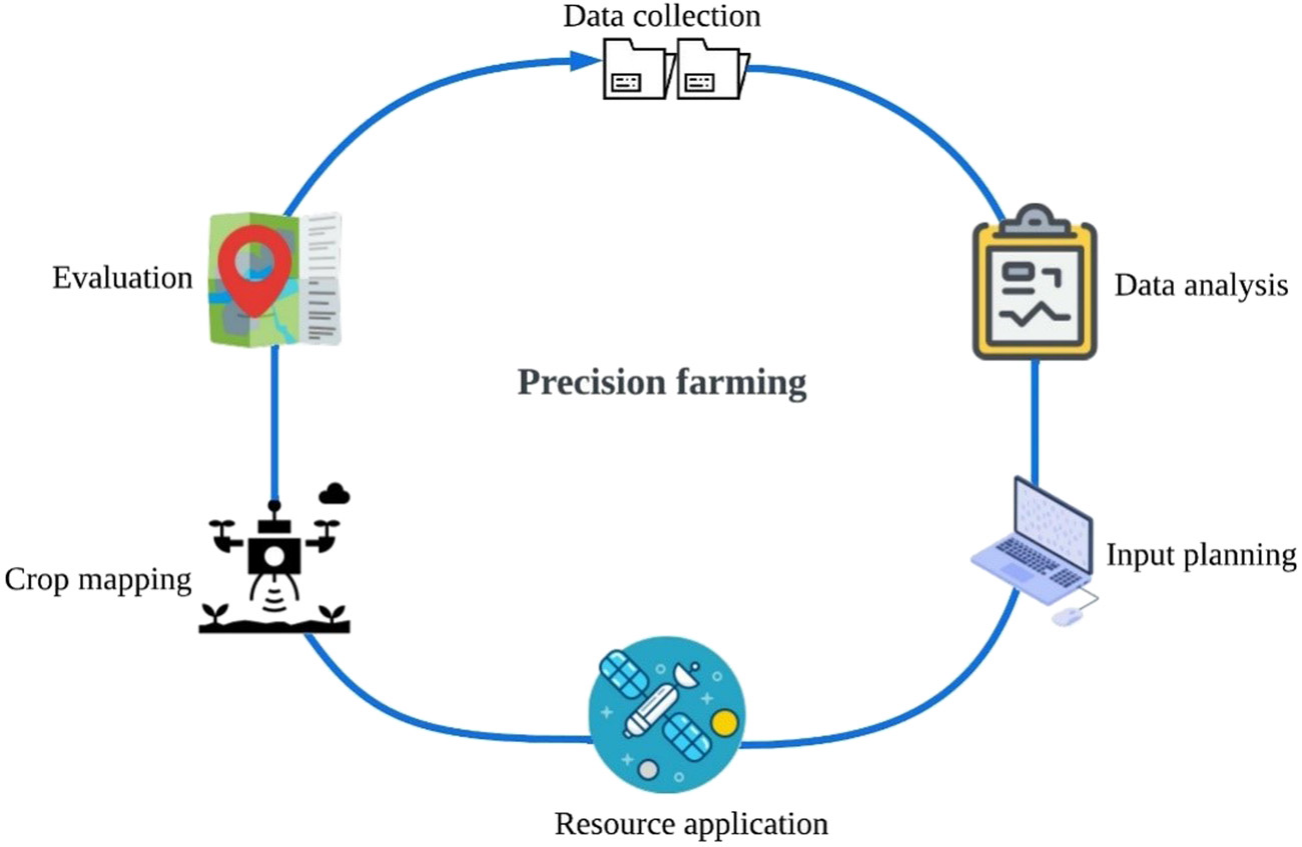
Proposed methodology of PF.

The methodology of the proposed PA model is depicted in Figure 4. In agriculture, PF has primarily focused on using extremely precise information of soil, weather, and crops to determine the appropriate fertilisers, pest control strategies, and even irrigation requirements. Resources can be employed in the “right quantities” and produce the “right amounts” when they are applied at the “right time.” The process of gathering data and its application is also an essential process.

In the proposed methodology, the data are collected through multiple stages to meet several aspects of farmland and then smart decisions are executed for irrigation routines; application of nutrients, fertilisers and other chemical or biological agents; pest control strategies; harvesting times; supply chain management; and sustainable development [40,41]. The strategy of the proposed design is designed considering several points which are mentioned below:

- Reduction in chemicals, fertilisers, and seed costs.
- Increase in yields improvement in the quality of crop.
- Tracing food production process and guarantee food safety.
- Document how food was produced profitability and document compliance with environmental regulations.
- Accuracy at higher speed and less affected by weather.

The instruments required to achieve PA are satellite positioning system (GPS), sensors, devices, software, and soil-crop simulation models. The proposed model of PF/agriculture cycle mainly consist of three stages.

#### 3.1.1 Data collection

**Figure 5:**
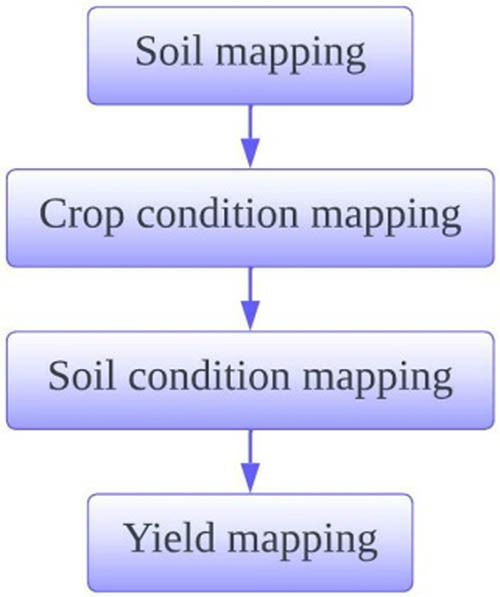
Data collection module.

Data collection is the measurement of within-field spatially variable soil and crop parameters and monitoring of local weather conditions. Data collection module of proposed model is depicted in Figure 5.

#### 3.1.2 Interpretation

**Figure 6:**
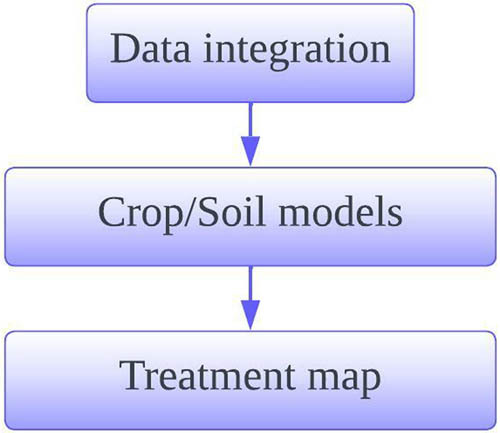
Data interpretation module.

Mapping of spatially variable rate crop input application. It consists of data integration, and creation of soil or crop models based on the input data. Data interpretation module of the proposed model is depicted in Figure 6.

#### 3.1.3 Application

**Figure 7:**
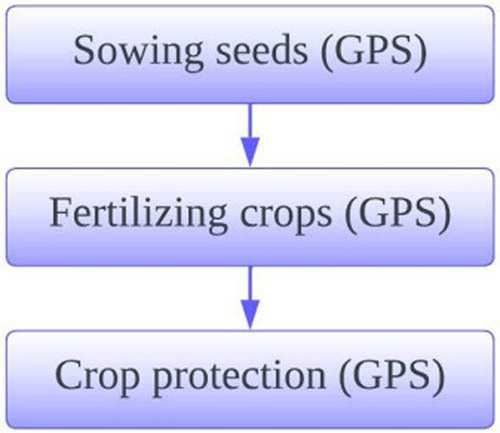
Application module.

It is spatially variable rate crop input application. The application includes GPS based sowing, fertilising, and protecting crops. Application module of the proposed model is depicted in Figure 7.

### 3.2 Proposed model of PA

**Figure 8:**
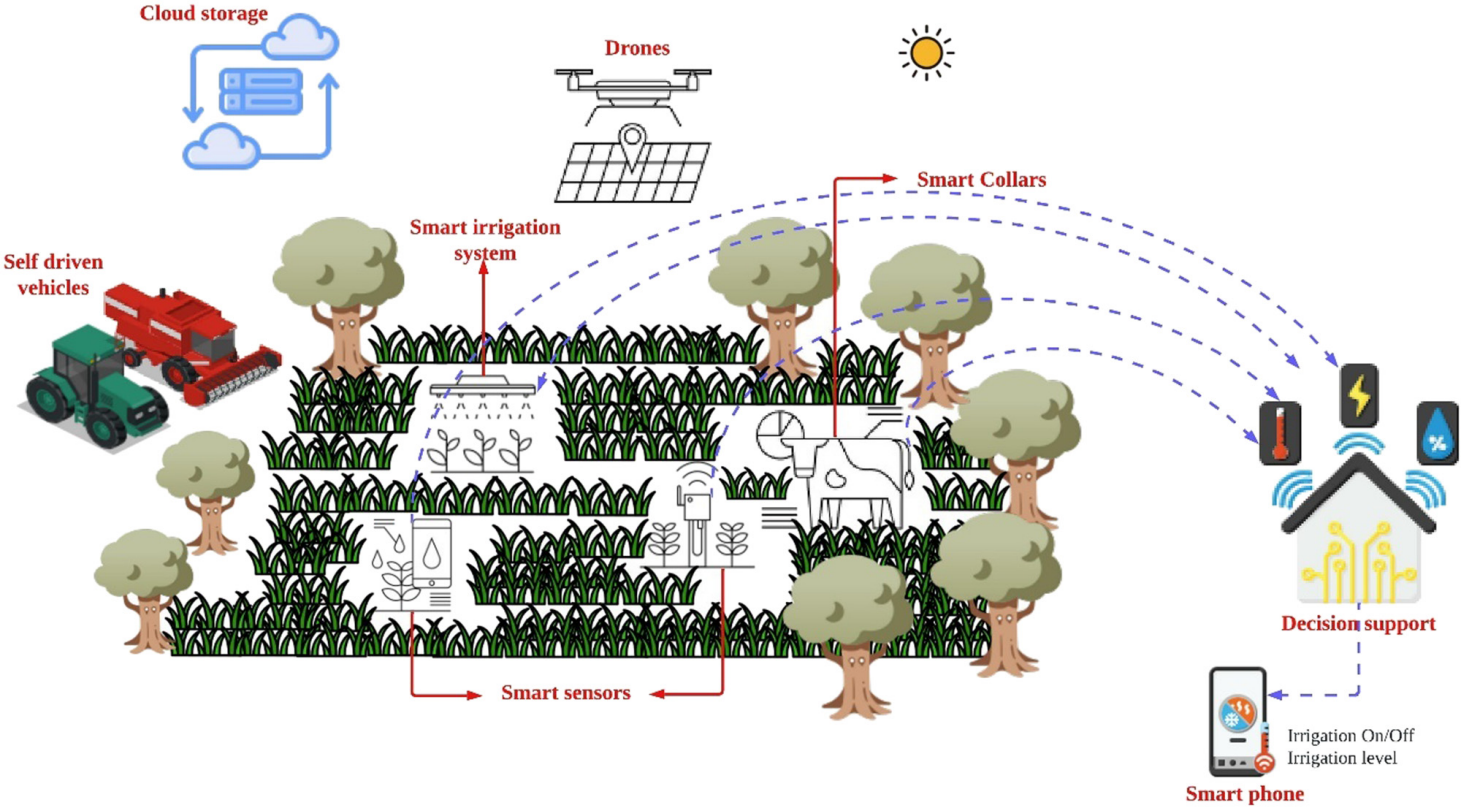
Proposed model of smart PA.

In agriculture, AI and robotisation are used mainly to interpret field images and apply fertilisers and pesticides with surgical precision, or for dealing with weeds. On a farm, for instance, smart sensors will be deployed for getting most recent information from the field in real time. The proposed model is depicted in Figure 8. The working of proposed model is described below:

#### 3.2.1 Smart crop sensors

These sensors will collect data about soil moisture, lack of nutrients, or the presence of pests to determine the requirement of irrigation, pesticides, or fertilisers.

#### 3.2.2 Smart irrigation system

In the proposed model, the irrigation system will work automatically to irrigate, fertilise, and fumigate each plot depending upon its individual needs, at exactly the right time with precise dosage.

#### 3.2.3 Smart collars

These smart collars on cattle’s and other animals will send important information about their health and other relevant information which may be of good interest. Other information such as, when an animal is ready to give birth or finding if any animal is sick.

#### 3.2.4 Drones

The drone will fly over the mapped area or the field, locating weeds, sick animals, and pathogens. These drones will also collect information about the development of crops (such as sugarcane in our case) and their needs.

#### 3.2.5 Cloud storage

The cloud storage will provide all necessary informational access to farmers or growers about their farm from all smart equipment and to optimise every stage of the production process.

#### 3.2.6 Self-driving vehicles

These vehicles will sow plant seeds with precision and at exactly the right depth and separation for the type of crop, maximising the output of the land.

#### 3.2.7 Decision support

It is an application that will be used for improving the decision-making capabilities. This system is responsible for analysing large amount of data and presents the best possible solution.

#### 3.2.8 Smart phone

This facility will provide time to time important information about the field data to farmers or growers. It consists of an application which will provide the controls for individual about turning On and Off the process of irrigation and also adjusting the level of irrigation.

### 3.3 Process flow and cycle of the proposed model

**Figure 9:**
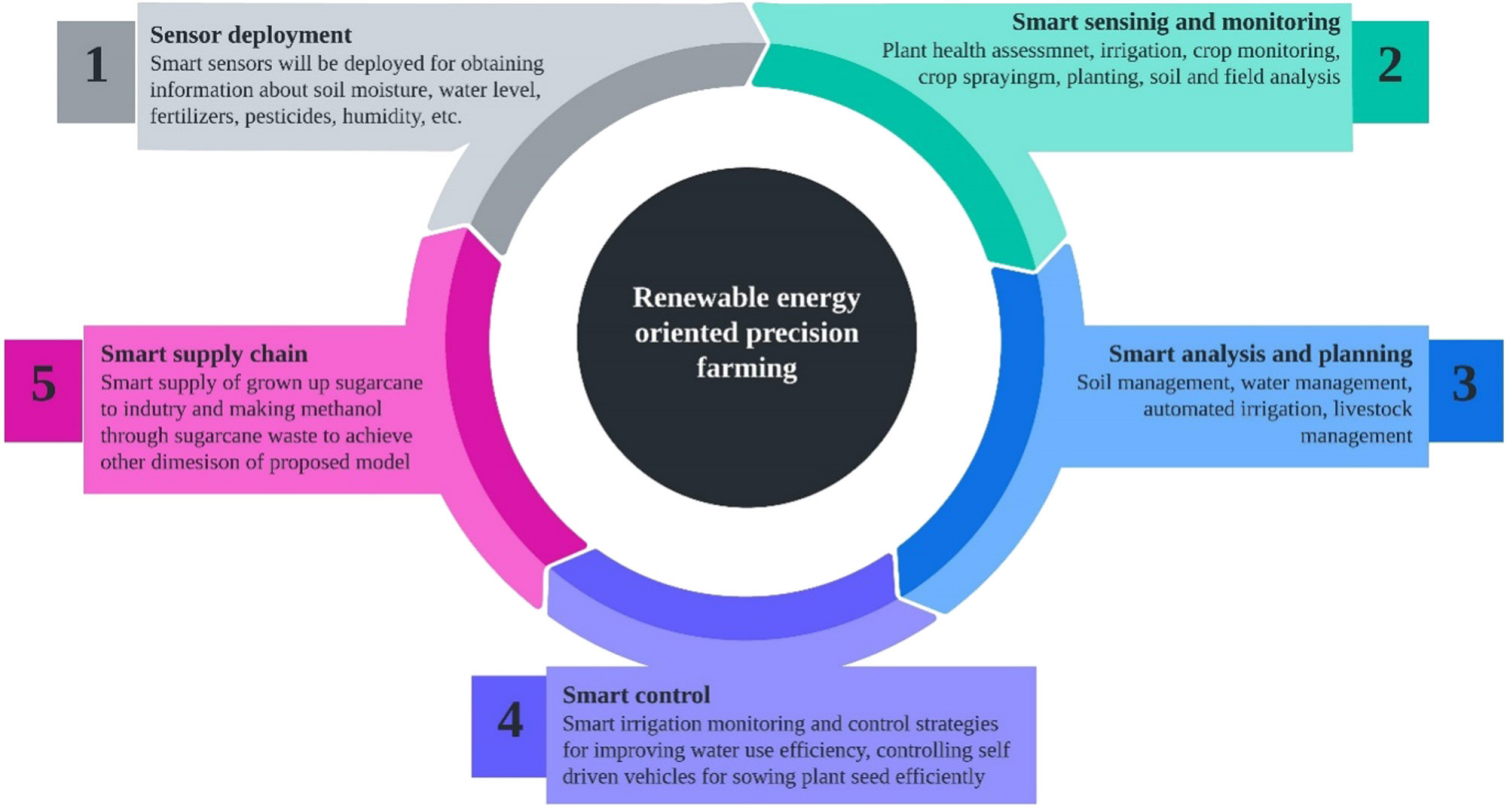
Renewable energy-oriented PF cycle.

In this section, working cycle and process flow of the proposed model of PA are discussed. The working cycle is depicted in Figure 9. The first phase of design cycle is sensor deployment where smart sensors are deployed for gathering information about soil moisture, water level, pesticides, fertilisers, temperature, and humidity. The second phase of the working cycle is smart sensing and monitoring of the agricultural field. In this phase assessment of plant health, requirements of irrigation, planting, spraying, and soil field analysis are taken care. In the third phase, smart analysis and planning are conducted, where soil management, water management, automated irrigation, livestock management are planned. The fourth phase is smart control, where smart irrigation monitoring and control strategies for improving water use efficiency, controlling self-driven vehicles for sowing plant seeds efficiently are promoted. The last phase of cycle is smart supply chain where smart supply of grown-up sugarcane to industry is analysed and also making of methanol through sugarcane waste to achieve another dimension of the proposed model is analysed.

**Figure 10:**
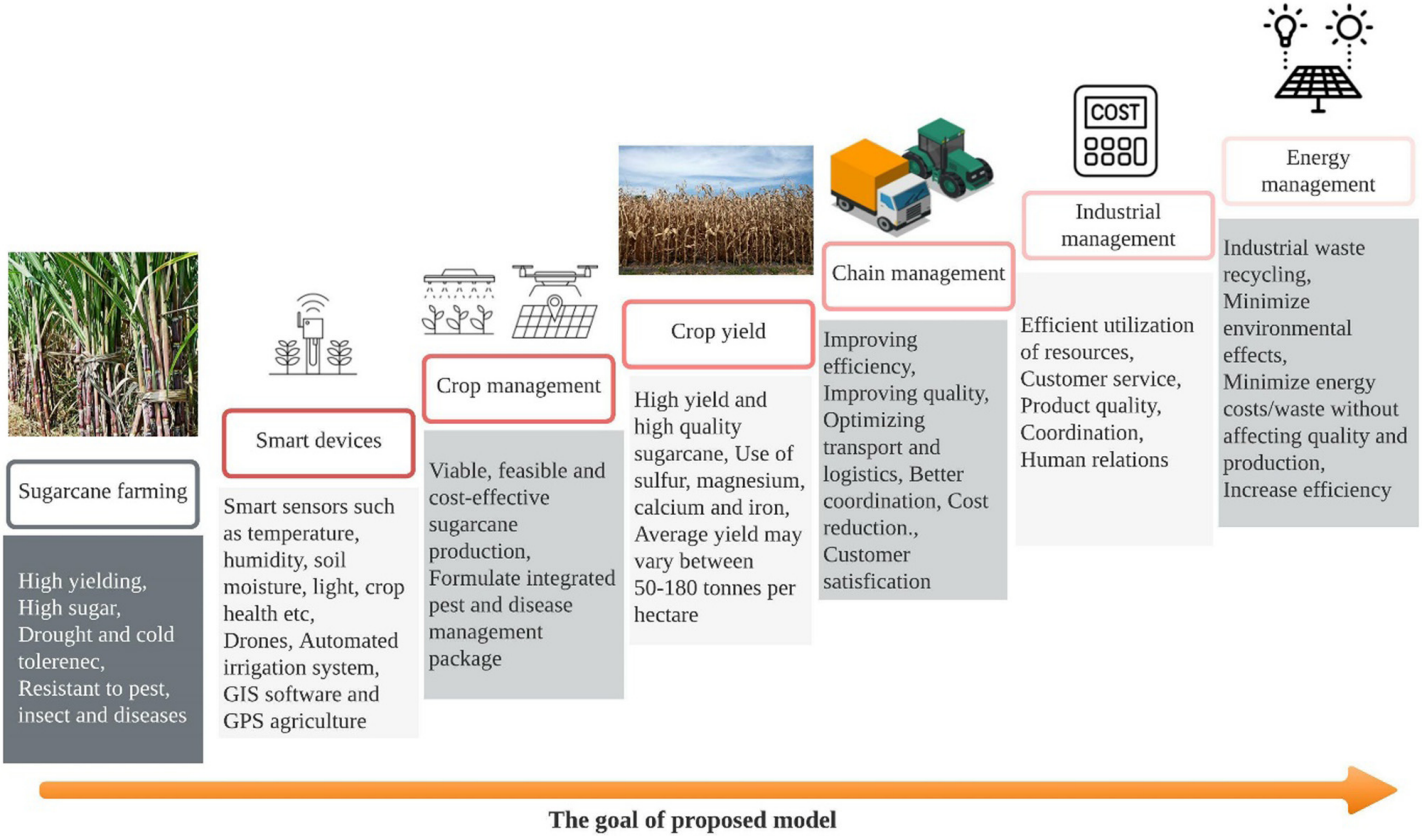
Process flow of the proposed model.

The complete working cycle or the process flow of the proposed design is depicted in Figure 10. The first phase of the proposed model is farming of sugarcane which is followed by the deployment of smart devices in agriculture field. In the next phase of process flow, group management is processed where cost-effective and feasible decision making are considered for the production of sugar cane. In the next phase, high-quality sugarcane is yielded by making use of sulphur, magnesium, calcium, and iron. It is supposed to achieve average yield between 50 and 200 tonnes per hectare. The next phase is supply-chain management which is focused on improving the efficiency and quality without compromising the optimisation of transport and logistics in order to achieve better coordination, cost reduction, and customer satisfaction. The next phase of process flow is industrial management where the efficient utilisation of resources considering customer service, product quality, human relations, and their coordination is designed. The last phase of process flow is an energy management which is focused on recycling of industrial waste, minimising environmental effects without affecting the quality of production to increase the efficiency of the product.

### 3.4 Sustainability and PA

**Figure 11:**
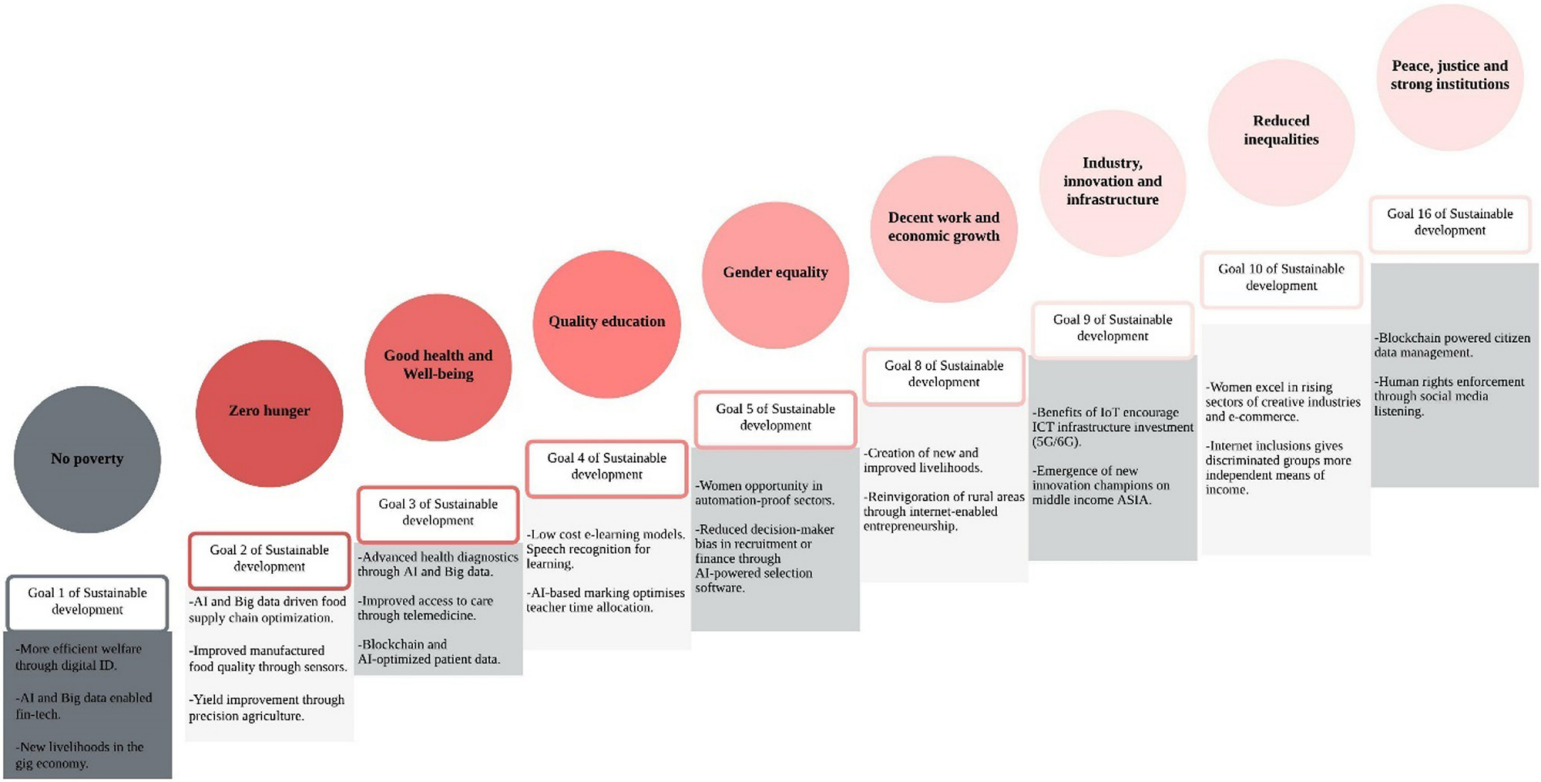
SDGs through PA.

Sustainability refers to agricultural and industrial technologies that reduced or prevented the environmental degradation often associated with economic activity. Sustainability is the ability to maintain the constant consumption or productivity by substituting between natural resources and manmade capital in production. Sustainable development goals (SDGs) through PA are depicted in Figure 11. There are 17 goals of sustainable development for the world transformation and agriculture can play an important role for meeting most of the SDGs. Smart sensing technology, management information systems, developments in farm equipment, and suitable economic and agronomic models have all contributed to the rise of PA in recent years. Precision agricultural practises improve outcomes like crop output and animal performance while also cutting costs and making the most of available resources.

Goal 12 of sustainable development is responsible consumption and production. The proposed model of PA is to address the SDG 12 also. The proposed model will provide consumers with information that encourages greater accountability. It will reduce waste through improved decision-making throughout the supply chain, aided by improved harvest yield and quality forecasting and planning. This model will reduce storage waste by enhancing planning and integrating agricultural sensors with transport management systems to reduce food deterioration. Additionally, the proposed model will reduce the use of chemicals and enhance long-term soil management via improved crop rotation planning.

## 4 Results and analysis

**Figure 12:**
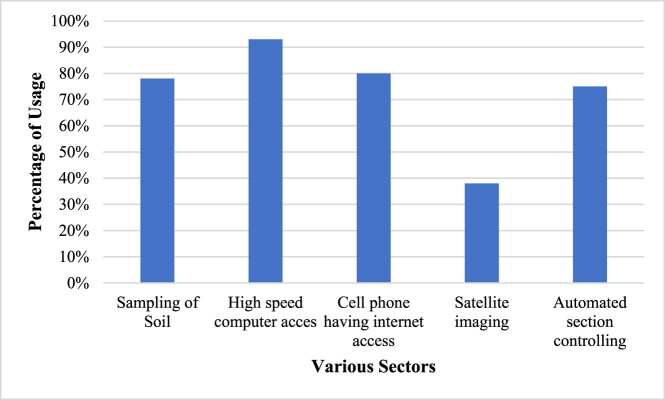
Usage of PA technology.

Farming prediction and effective decision-making are enabled through real-time data acquisition, and empirical analysis is achieved through mathematical simulations by implementing graph neural networks. Experimentation is conducted on crops utilizing scientific facts, practical knowledge, and technical artifacts, leading to the development of the proposed model and contributing to interdisciplinary research in science and technology. As per the joint collaboration with Russia, the farmers of both the countries will be benefitted utilising real-time crop monitoring and tracking. The mass reachability will be the key point of this exchange with Russia, utilising mobile application and AI platform to reach wide segment of researchers, environmentalists, farmers and agricultural economists, etc. Productivity will be enhanced for making in-field decisions based on PF. The outcomes of this research work will correspond to the world level and are aimed at developing the PF technology for mass reachability of farmers. The project responds to global challenges in the field of agriculture by creating knowledge transfer and farming prediction. The technological outcomes will be utilising the transition of personalised deep learning; transition to a quantitative assessment of the farming and agricultural process based on real-time data analysis, etc., taking into account the explosive growth of engagement in agriculture. PA technology has been adopted in various areas and its usage has been popularised in different sectors including sampling of soil, high speed computer access, cell phone having internet access, satellite imaging, automated section controlling, etc. The percentage of PF adoption by several sectors is presented in Figure 12.

**Figure 13:**
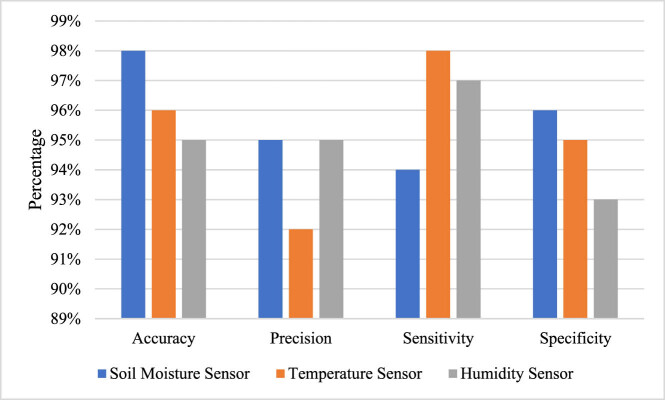
Performance evaluation of IOT sensors.

Various PA technologies enable users to harness the research potential for constructing several variables such as soil type, weather conditions, computer access, and real-time monitoring. Further, the PF practices are being used to provide the solutions for overcoming the challenges of raising the climatic variations, inadequate weather conditions, soil conditions, and reduction in waste and greenhouse effect. The PA is important to check the natural disaster conditions and agricultural production effects. Further, various sensors are used with IoT aspect, for sensing the soil and nutrient content of the PA. The outcomes of sensitivity, specificity, precision, and accuracy are obtained for various IOT sensor data, based on true positive, true negative, false positive, and false negative events, which are depicted in Figure 13. Various sensors are utilised for this experimentation, including soil moisture sensor, temperature sensor, and humidity sensor. They are utilised in IOT sensors in order to check the reliability of PF concept. It reveals monitoring of crops in order to promote PF with enhanced security and reliability.

Further, in PF, the selection of an appropriate classifier plays a vital role in achieving accurate and reliable predictions. To compare the performance of different classifiers, namely, naive Bayes, k-nearest neighbours (kNN), random forest, and the proposed method, an evaluation based on accuracy can provide valuable insights. The benchmark dataset utilised in this research for classification analysis of precision framing is FAOSTAT1
https://www.fao.org/faostat/en/#home
. FAOSTAT is a database maintained by the Food and Agriculture Organisation of the United Nations. It offers a wide range of agricultural statistics, including crop production data, fertiliser usage, and land use statistics, which can be valuable for PF analysis. FAOSTAT provides access to agricultural statistics from countries all over the world, including crop production data, livestock production data, trade statistics, fertiliser usage, land use statistics, and food balance sheets. The database allows users to explore and download data, create custom queries, and generate reports and visualisations. With its extensive collection of agricultural data, FAOSTAT enables researchers, policymakers, and practitioners to gain insights into global agricultural trends, assess crop performance, analyse production patterns, and make informed decisions in the context of PF. The benchmark of FAOSTAT dataset includes typical scenarios used in PF including soil qualities, weather patterns, crop traits, and other pertinent factors.

**Figure 14:**
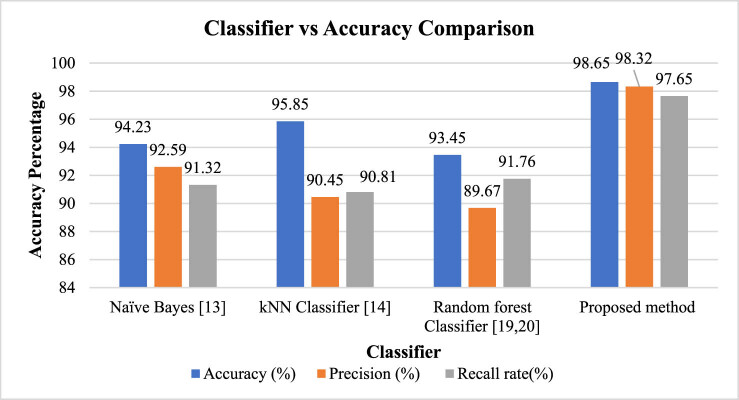
Classifier vs accuracy comparison for PF.

The comparison of various classification approaches in terms of accuracy is drawn in Figure 14.

The comparative analysis utilising FAOSTAT benchmark dataset reveals that naive Bayes classifier is extensively used in many different fields and is based on the idea of feature independence. Fast training and prediction times are available. However, it could have trouble with intricate interactions between variables. The non-parametric classifier kNN, on the other hand, places a new sample in the same category as its closest neighbours. The number of neighbours (k) and distance measure that are used have a significant impact on the accuracy of the method. Although kNN is simple to use, it can be costly to compute for big datasets. In order to increase accuracy and manage complicated interactions, random forest is an ensemble classifier that mixes several decision trees. It has resilience against overfitting and effectively handles big datasets. The model’s interpretability could be compromised by its ensemble nature. The proposed method involves certain PF-related steps and it outperforms various state-of-the-art classification methods providing 98.65% accuracy, 98.32% precision, and 97.65 recall rate for classification analysis of PA utilising FAOSTAT benchmark dataset. By comparing its accuracy to that of the other classifiers described above, its performance shows better outcomes and is reliable. The evaluation of the suggested technique is essential to ascertain its efficacy and competitiveness since it attempts to use developments in AI, ML, and IoT to improve PF outcomes.

**Figure 15:**
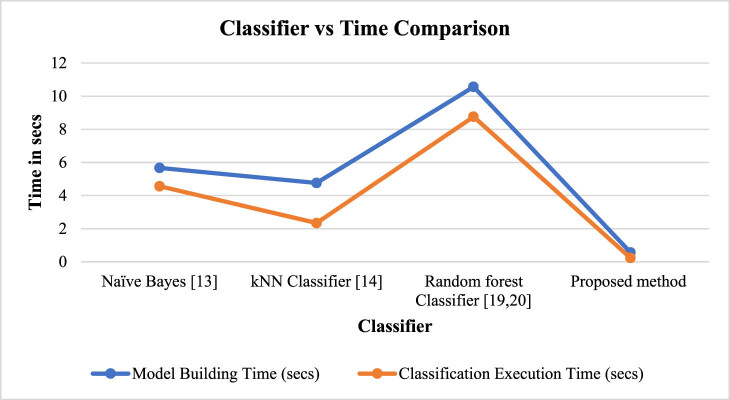
Classifier vs execution time comparison for PF.

Other than accuracy, execution time of model building and classification is an important consideration for PF applications which is compared in Figure 15.

The graphical representation reveals that the naive Bayes’ fundamental probabilistic methodology often results in quick model construction and prediction times. It is computationally effective since it computes probability based on feature independence. However, lengthy feature engineering or data preparation may make the model construction process take longer. Since all that is required to develop a kNN model is to store the training data, it is quick. Due to the creation of several decision trees, random forest model generation can take longer than naive Bayes and kNN. The specific methods and algorithms used in the proposed method will have an impact on how quickly the suggested method can construct models and make classification decisions. Model development and classification execution times may be competitive if the suggested strategy effectively integrates cutting-edge AI, ML, and IoT technologies. It is feasible to analyse the computing efficiency and viability of several methods for real-time PF applications by contrasting the model construction and classification execution times of naive Bayes, kNN, random forest, and the suggested approach. In situations involving PF, the proposed method gives a considerable advantage over conventional classifiers if it provides competitive execution time of 0.23 s while retaining high accuracy.

## 5 Discussion

The findings and analysis of this study demonstrate the important contributions made by ML, AI, empirical analysis, real-time data collection, and PF in a variety of fields. PF technologies have been developed and optimised via multidisciplinary cooperation and experimentation that combines scientific knowledge and real-world observations. The combined research effort with Russia, which seeks to assist farmers in both nations through real-time crop monitoring and tracking, is a crucial component of the study. The usage of mobile apps and AI platforms enables extensive interaction between academics, environmentalists, farmers, and agricultural economics. PF has the ability to completely transform agriculture on a global scale by increasing production and facilitating in-field decision-making. The results of this research do not just have popular appeal in mind, but also have the development of precision agricultural technology that meets worldwide standards. This study responds to the increased interest in agriculture and advances information transmission and agricultural forecasting by utilising personalised deep learning, real-time data analysis, and quantitative evaluations. Several industries have already embraced precision agricultural technology. PF has gained popularity across a variety of industries thanks to the use of techniques including soil sampling, high-speed computer access, satellite imagery, and automated section management. These innovations allow for the collection of information on past production, such as soil type, weather patterns, computer access, and satellite controls, which expands the scope for study and analysis. Therefore, PF techniques provide answers to problems brought on by climatic changes, poor weather, soil conditions, waste reduction, and greenhouse impacts. It is essential for tracking how natural catastrophes affect agricultural productivity. The use of IoT sensors in PA makes it possible to monitor soil and nutrient levels. This information, together with assessments of sensitivity, specificity, precision, and accuracy, offers insightful information on the dependability and security of PF techniques. To verify the accuracy of PF principles, a variety of sensors, including soil moisture sensors, temperature sensors, and humidity sensors, have been used. These sensors support precision agricultural techniques with increased security and dependability and contribute to improved monitoring capabilities.

The PF classifier comparison and execution time study emphasis the significance of choosing the best classifier for precise forecasts and effective decision-making. The performance and possible applicability of naive Bayes, kNN, random forest, and the suggested approach are compared, and the results are quite insightful. The examination of model construction and classification execution times offers information on the computational efficiency of the classifiers while accuracy assessment indicates which classifier produces the most accurate predictions. Farmers and researchers can choose the best classifier to utilise in PF applications by carefully considering execution time and accuracy. The suggested approach, which combines cutting-edge innovations and technology created especially for PF, has the ability to improve accuracy and provide competitive execution times. Its evaluation and comparison against traditional classifiers serve as a crucial step in assessing its effectiveness and determining its suitability for widespread adoption in the field. It further emphasises the significance of selecting classifiers that strike a balance between accuracy, efficiency, and the specific requirements of PF, ultimately contributing to more sustainable and productive agricultural practices.

The findings of this study demonstrate how PF has the potential to transform worldwide agricultural practises. PF provides prospects for higher production, resource optimisation, and environmental sustainability by combining cutting-edge technology, real-time data analysis, and ML algorithms. For precision agricultural practises to improve and become widely used, cooperation between academics, politicians, and farmers is crucial. The findings and analyses offered in this study show the value and effectiveness of PF in resolving the issues faced by the agricultural industry. PF may increase output, optimise resource management, and reduce environmental impacts by utilising real-time data, cutting-edge technology, and multidisciplinary collaborations. PF techniques are made even more dependable and secure with the use of IoT sensors. To fully realise the promise of PF and ensure its widespread adoption for the benefit of farmers and the agricultural sector as a whole, it is essential to continue research, innovation, and information exchange.

### 5.1 Limitations and future recommendations

While PF offers many advantages and breakthroughs, there are certain restrictions to take into account. It is critical to discuss these shortcomings and offer suggestions for further advancements.

#### 5.1.1 Cost Barrier

The initial setup expense of implementing PF can be a major barrier for small-scale farmers and is one of the main hurdles. Governments and agricultural organisations should offer financial assistance, subsidies, and other rewards to promote the broad use of PF techniques in order to get over this barrier. Continued research and development initiatives should also concentrate on creating affordable technologies and solutions that are suited for the requirements of small-scale farmers.

#### 5.1.2 Technological infrastructure

A strong technology infrastructure, including dependable internet access, data storage options, and sensor networks, is essential for PF. Poor infrastructure in certain rural regions may make it difficult to apply precision agricultural methods effectively. In order to increase connection and infrastructure in rural agricultural areas and give farmers access to the resources they need for effective implementation, governments and technology providers should work together.

#### 5.1.3 Data privacy and security

Data privacy and security are issues that are brought up by PF’s extensive data collection and use. It is essential to set up stringent data exchange procedures, secure data storage systems, and strong data protection mechanisms. To protect farmer data and handle possible privacy and security issues brought on by precision agricultural technology, governments and regulatory agencies should create rules and legislation.

#### 5.1.4 Accessibility and education

Although PF has many advantages, its effective use necessitates a certain degree of technological knowledge and proficiency. Farmers should have easy access to training programmes, workshops, and instructional materials on PF to ensure that they have the knowledge and abilities to efficiently utilise cutting-edge technologies. PF practises may be encouraged and spread through partnerships between agricultural colleges, extension agencies, and industry players.

#### 5.1.5 Integration and interoperability

As precision agricultural technologies advance, it is more important to guarantee compatibility and interoperability across various systems and gadgets. Prioritisation should be given to standardising data formats, communication interfaces, and protocols to allow for easy information interchange and integration across various platforms. The creation of open-source platforms and interoperability frameworks may be accelerated through industry alliances and efforts, fostering a more united and integrated PF strategy.

#### 5.1.6 Sustainability and environmental impact

Although PF strives to reduce its negative effects on the environment, it is crucial to regularly monitor and assess its sustainability practises. Within PF frameworks, more research and development initiatives should concentrate on improving resource management, lowering greenhouse gas emissions, and fostering regenerative agriculture practises. Precision irrigation systems, organic farming techniques, and the incorporation of renewable energy sources may all help ensure the long-term viability of PF.

While the use of PF offers considerable improvements in agricultural methods, several issues need to be resolved in order for it to be widely adopted and reach its full potential. PF may benefit farmers of all sizes by overcoming obstacles including financial constraints, lack of technology infrastructure, data privacy concerns, and accessibility issues. A sustainable and effective future for agriculture will be enabled by ongoing research and development, stakeholder cooperation, and innovation.

## 6 Conclusion

Modern agriculture has undergone a paradigm shift as a result of PF, which has completely changed how decision-makers and resource managers operate. PF helps farmers to make correct and timely choices by combining cutting-edge technology like AI, ML, and IoT, which increases production, lowers costs, and has a smaller environmental effect. The proposed PF approach is created with the intention of saving farmers money and time. Farmers may streamline operations and boost overall productivity by using AI, ML, and IoT technologies to automate data collection, analysis, and decision-making processes. PF’s base is built on the gathering and structuring of data, but its actual potential rests in the use of diverse technologies to convert the collected data into efficient management techniques. The proposed model of PF seeks to lower the cost and environmental effect of agricultural practises, by maximising the use of pesticides and fertilisers. Farmers may use fewer chemicals, reduce pollution, and improve sustainability in general by utilising data-driven insights and precise application strategies. PF delivers long-term benefits that exceed the disadvantages despite the upfront expenses, offering a dependable and scalable RoI. The main advantages of PF are that it can be used in a variety of agricultural activities and landholdings, making it practical for farmers of various sizes. The proposed methodology can increase crop yields while drastically reducing the quantity of fertilisers and other agricultural inputs utilised. Farmers may maximise their usage of water, fertilisers, and pesticides to increase their RoI while preserving the environment, which may also reduce mistakes and increases production. Thus, PF represents a paradigm change in agriculture, enabling farmers to make knowledgeable decisions based on current information and cutting-edge technology. The proposed approach encompasses a series of PF-oriented procedures and surpasses various state-of-the-art classification methods in terms of performance. It attains a remarkable classification accuracy of 98.65%, accompanied by a precision of 98.32%, and a recall rate of 97.65% while retaining competitive execution time of 0.23 s, when applied to the analysis of PF utilising the FAOSTAT benchmark dataset. The suggested methodology offers several advantages by combining AI, ML, and IoT, including enhanced profitability, improved resource management, and increased agricultural yields. PF has the potential to revolutionise agriculture and influence the development of sustainable farming methods in the future as technology and research in this area evolve. Farmers may help the agriculture sector become more effective, productive, and ecologically sensitive by adopting this unique strategy.
